# Exploring online problem gamblers' motivation to change

**DOI:** 10.1016/j.abrep.2019.100187

**Published:** 2019-06-17

**Authors:** Ayna B. Johansen, Pål Fylling Helland, Dag K. Wennesland, Edmund Henden, Håvar Brendryen

**Affiliations:** a Norwegian Centre for Addiction Research, University of Oslo, Norway; b Blue Cross Addiction Treatment Center, Oslo, Norway; c Centre for the Study of Professions, Oslo University College, Norway

**Keywords:** Motivation, Problem online gambling, Online treatment referral, Projection

## Abstract

**Background:**

In order to improve interventions for problem gambling, there is a need for studies that can highlight psychological factors that support the desire to reduce gambling.

**Objective:**

To explore online problem gamblers' motivation for change by studying participants' reactions to an online treatment referral website designed to motivate at-risk gamblers to seek help.

**Design:**

A qualitative evaluation study, combining focus groups and in-depth interviews. Data were analyzed using the general inductive approach.

**Informants:**

The informants included 19 male, treatment- and non-treatment seeking, online gamblers who played a variety of games, including poker, sports betting and online casino.

**Results:**

Motivation to change emerged as two processes including (a) empathy with others, which included projection of their thoughts and feelings onto others, and (b) dissonance between gambling behavior and ideal self-image. Dissonance included two subthemes: (i) dissonance due to positive feelings towards sports and athletics, and (ii) dissonance due to gambling among family.

**Conclusions:**

The findings have implications for interventions designed to evoke motivation early in treatment of online problem gambling. Inducing problem gamblers to reflect on the thoughts and feelings of concerned significant others, real or fictional, could be a viable strategy to motivate online problem gamblers to consider change.

## Highlights

• We identified three sources of motivation for changing gambling behavior:
• Empathy with concerned significant others, real or fictional
• Cognitive dissonance due to positive feelings towards sports and athletics
• Cognitive dissonance due to gambling among family members

## 1 Introduction

Most problem online gamblers go undetected by the treatment system, which puts them at risk for a range of negative outcomes, including suicide (Wong, Kwok, Tang, Blaszczynski, & Tse, 2014). Due to a proliferation in online gambling opportunities (Gainsbury, 2015) and aggressive and personally targeted advertising, the online contexts of many at-risk and problem gamblers strongly support continued gambling.

Problem gambling can cause financial difficulties (Downs & Woolrych, 2010), crime and legal problems (Hodgins, Peden, & Cassidy, 2005), increased conflict within relationships (Downs & Woolrych, 2010), work-related problems (Holdsworth, Nuske, & Hing, 2013), psychological distress (Lorains, Cowlishaw, & Thomas, 2011), substance abuse problems (Lorains et al., 2011; Welte, Barnes, Wieczorek, Tidwell, & Parker, 2004) and increased risk of suicidality (Ledgerwood & Petry, 2004; Wong et al., 2014). These factors interact in creating a situation where individuals continually increase their gambling in an attempt to either escape negative feelings or to increase income (Holdsworth et al., 2013; Mann et al., 2017).

Given the seriousness of the issues faced by problem gamblers there should be no shortage of reasons to change their gambling behavior. Yet many remain trapped in the addictive cycle of gambling: negative feelings associated with gambling losses, combined with the positive experience of the gambling activity itself, a shortage of money, and the need to keep the extent of gambling hidden from others (Orford, 2013). There is a substantial psychological literature on problem gambling in terms of motivations to gamble (Wood & Griffiths, 2007), stages of change (Kowatch & Hodgins, 2015), and change processes in general (Kushnir, Godinho, Hodgins, Hendershot, & Cunningham, 2015). Studies surveying help-seeking problem gamblers have shown that reasons for seeking help are primarily crisis-driven rather than a result of a gradual process of becoming motivated (Evans & Delfabbro, 2005). In light of addiction research generally showing that a large percentage of people recover without professional help, it is possible that reasons for help-seeking are different from reasons for becoming motivated to change gambling behavior (Klingemann, Sobell, & Sobell, 2010).

Self-discrepancy theory is relevant to understanding the process of becoming motivated to change, as it relates to exploring dissonance (Higgins, 1998). This theory posits that the perceived difference between actual and ideal selves produces feelings of shame, internalization, and depression, while the difference between actual and ought selves, or the idea of what one should be to another person, relate to feelings of guilt and agitation. The idea of dissonance as being a source of motivation is also found within Motivational Interviewing (MI), where helping the person explore discrepancies between actual behavior and their values within a supportive, non-confrontational conversation is theorized to motivate change (Miller & Rollnick, 2013). Direct confrontation (i.e. the therapist pointing out such discrepancies) is thought to be less helpful and create unnecessary resistance, due to triggering strong feelings of shame and guilt as well as psychological reactance, an unwillingness to be coerced or told what to do. Bjerg (2010) found that problem gamblers who prefer games of skill are put off by traditional treatment approaches, where gambling is viewed as resulting from cognitive distortions (Ladouceur, Sylvain, Boutin, & Doucet, 2003).

To our knowledge no study has examined the motivation for change of problem gambling qualitatively from the individuals' perspectives. These factors can have implications for treatment in terms of how one approaches individuals. More flexible services, informed by knowledge of the sources and processes of building motivation for change of problem gambling, may increase treatment-seeking of individuals with problem gambling, and decrease treatment dropout. Thus, to identify factors related to gamblers' motivation for change, this study explored the process of becoming motivated to change, including sources that trigger dissonance (Higgins, 1998), defined as the difference between actual and ideal selves.

## 2 Methods

To learn more about both the sources of motivation as well as the process of becoming motivated to change among online gamblers, we conducted a qualitative study. Data was analyzed using the general inductive approach (Van Manen, 1990). This is a method where researchers and participants interact to describe and interpret an experience from the participant's point of view.

### 2.1 Sample

We recruited a purposive sample of 19 male online gamblers that ranged in age from 20 to 44 years of age, with a median age of 30.5, and a mode education of 2 years of college. Fourteen lived with either a partner or children, but six were not in a committed relationship. Three of the participants were in long-term treatment, five were treatment seekers, and 11 were non-treatment seekers. Fourteen participated in four focus groups, and five provided individual, in-depth interviews. All reported gambling online as their preferred environment, although most reported also gambling in other environments. Poker and sports betting was the preferred games among the skill-based games, and online slot machines for the non-skill based games.

### 2.2 Setting and procedure

The study was conducted in cooperation with an outpatient addiction treatment center run by Blue Cross Norway, a non-profit organization conducting an early intervention program for young male problem gamblers. The treatment center had developed a referral website to recruit gamblers for treatment. Online gamblers were invited to help evaluate the referral website, and the website was used as stimulus material during data collection. The webpage includes a self-test, written information on gambling and problem gambling, and video interviews with fictional gamblers and their significant others. The videos illustrated three men with different gambling experiences and levels of motivation for change, and one girlfriend who discussed her ambivalence about living with someone who gambles too much.

The three participants that were in long-term treatment was recruited from the outpatient clinic, and the five treatment-seeking participants were recruited after having engaged with the referral website and subsequently received one or two sessions of motivational interviewing. The final 11 participants were recruited through posts on two Internet forums for gamblers.

The individual interviews were conducted by an experienced clinical psychologist or a psychology graduate student and conducted at the research facility, or the informant's workplace or home. The focus groups were run by two experienced clinical psychologists. Focus groups first examined participants' preferred games and modes of gambling. Then the focus group moderator focused the discussion on features of the referral website, examining issues such as face validity/professionalism, user friendliness, and personal relevance. For each type of stimulus (e.g. self-test, written information or video-interview) the moderator also examined participants' perceptions of the presentation and framing in terms of relating to their own motivation to change gambling, at which point the moderator allowed the group members to develop themes and responses without interruption. As such, the focus groups developed from a moderated group interview to being a true focus group, where group members would interact (Belzile & Öberg, 2012). This included commenting on each other's point of view, and even challenging each other's motives (Kidd & Parshall, 2000).

### 2.3 Analytic procedure

The naturalistic context of producing a problem gambling referral website led us to choose the general inductive method, which is appropriate for theory-building in applied fields (Lynham, 2002) and the analysis of qualitative evaluation data (Thomas, 2006). The method helps establish links between the evaluation and summary findings derived from the raw data and develops a model of the underlying structure of experiences or processes evident in the raw data. Similar to grounded theory, it can be used to produce a model, but the goal is generally to produce a holistic analysis that includes both descriptions (themes) and explanations (processes). As applied in the current study, this analytic strategy limits theory-building to the presentation and description of the most important categories and befits qualitative investigations based on evaluation data because the identification of unplanned or unanticipated effects are seen as important as evaluating the expected (Thomas, 2006). Focus groups and interviews were digitally recorded and transcribed verbatim. We used two coders to analyze the data, a research assistant and the first author. The transcripts were reviewed several times, by both coders, looking for statements describing events and persons that initiated problem awareness and motivation for change. Through reviewing and discussing the participants' descriptions, we tried to clarify the “essence” of these experiences, and cluster the statements into relevant themes.

We looked for events and persons that initiated dissonance (Higgins, 1998), problem awareness and motivation for change. We defined motivation for change as “change talk” (Miller & Rollnick, 2013), which are expressions of desire, ability, reasons and needs for change, as well as defensiveness or ambivalence, which resolution can initiate motivation. To maintain analytic rigor we maintained a written audit trail that included field notes, methodological considerations and analytic notes (Rodgers & Cowles, 1993). Both interview and focus group guides contained open-ended questions about the stimulus materials and motivation for change of gambling habits. The first three focus groups used the referral website as stimulus materials and points of discussion. Two groups contained mainly non-treatment-seeking persons, and one group including treatment-seeking persons. Participants were encouraged to talk about their personal histories when explaining their opinions on the stimulus material. Individual interviews were conducted with individuals who had utilized the website for referral, and also here it was used to stimulate discussion within the interview itself. Emerging themes from the focus groups were explored within the interviews. The initial focus group data reflected a general understanding of the range of motivations and contributed to developing preliminary themes that were subsequently used to guide the exploration of the phenomena in the individual interviews. A fourth and final focus group was eventually completed to extend the findings. This group verified the existing results without adding new information, representing a point of acceptable data saturation.

### 2.4 Ethical considerations

The study was approved by the Norwegian Regional Ethics Committee (Reference number 2011/711), and all participants provided written informed consent, after being given time to consider it. Participants received gift cards worth 500 NOK (approx 65 USD) to compensate for time spent in interviews.

## 3 Results

### 3.1 Focus group first impressions

In reviewing the data from the initial focus groups, we found that all the non-treatment-seeking online gamblers were in consensus that their motivation to consider changing their gambling behaviors depended on a sense of being “out of control”, as evidenced by noting losses and betting recklessly, either by making bad plays in poker or by losing sensitivity to the size of bets made in non-strategic games.

### 3.2 Change themes and processes

Two change-related process themes were identified: (a) empathizing with others, and (b) dissonance between gambling behavior and ideal self-image. The latter included two sub-themes: (i) dissonance due to sports and athletics, and (ii) dissonance due to gambling among family.

1. *Empathizing with others*.

Some of the participants who were not already motivated to change gambling approached the behavior during the interviews by trying to understand what it might mean in the eyes of their concerned significant others. In some cases, the process involved participants creating their own stories about how a loved one might feel about the participants' gambling, empathizing with the feelings of the loved one, and using the resulting empathic feeling as motivation for change. When imagining qualities onto others in the context of confronting gambling problems, participants would compare themselves with others. For example, one participant who had been coerced into treatment by his mother reported becoming motivated to change during the research interview. Specifically, his motivation for change only became apparent when imagining being observed by the grandfather whom he envisioned emotionally hurting. The contents of the grandfather's attributions in one sentence evolved into expressing his personal motivation in the next. That is, he acknowledged his addiction through discussing that of his grandfather:

“*The guilt is the worst thing about it*. *No doubt*. *To disappoint people*. *To disappoint Grandpa*. *That was terrible*. *Grandpa*, *you know*! *He*'*s had some addiction problems as well*, *in his younger days*. *And he*'*ll probably get the feeling that it runs in the family and stuff like that*. *And I don*'*t want it to be like that*. *I want to try to keep it from other people* - *that it is their fault*. *Try to put it all on myself*, *that is all I really want*.”

Empathizing comments were also generated by non-treatment -seeking participants who watched the intervention videos displaying the responses of a gambler's girlfriend. The video demonstrated a young woman talking about the consequences of her boyfriend's gambling to her, and the video prompted the participants to start identifying her reactions and their meaning. In processing the feelings of the character in the video, it appears the gamblers experienced the same feelings as the woman talking, but nonetheless ascribed those feelings to her. The following quote illustrates the transitioning from external to internal motivation for change through accepting and processing the fictional girlfriend's experiences:

“*People who you care about*, *I think it hits harder than listening to someone who is a bit like yourself*… *who it is easy to distance yourself from anyhow*. *But a* [*hypothetical*] *girlfriend stepping on their toes may have more of an effect*… *if she says any of the same things that my wife complains about at home*, *and if* she makes any other valid points… well, I think that will hit home.”

2. *Dissonance between gambling behavior and ideal self*-*image*.

A main motivating factor in the decision to start treatment for the treatment-seeking participants and those already in treatment was awareness of problems related to the gambling. Dissonance would come from comparing financial problems, family/relationship problems, workplace issues, comorbid drug/alcohol abuse, signs of gambling addiction, and consequences to self-image, to how they felt things ought to be. Dissonance could also come from doing things one normally does not do, such as deceiving others or stealing. The greater the discrepancy between their experience of the current situation and the ideal self-image, the stronger and more unpleasant the feeling of guilt was. One man reported that the self-image dissonance came from being able to stop once, and then relapsing – thus projecting himself as through time. He explained that while he could excuse his first mishap as “*youthful stupidity*”, the second one “*was on him*”, implying that his behavior was now in contrast to his ideal self is one of being someone who acts rationally and learns from their mistakes. Some described the problem awareness as “*knowing they were at the end of their rope*”. Also, the respondents highlighted that they did not feel ‘normal’, meaning they felt irrational and different, as well as apart from other people. Participants often experienced several sources of dissonance at the same time, as one young man who came to recognize his loss of control, also registered loss of money and changes in his relationships at the same time:

“*One year ago I gambled away my entire paycheck in just*… *I even blew off work and just played the entire day*. *And then I didn*'*t have any money left for the whole month*, *so I had to lie to my parents*, *and it was such a blow*, *I knew it was a serious problem*, *because if you are normal*, *you would have a tiny bit of control and not gamble recklessly*. *So if you had control*… *a normal person would not have done such a thing*.”

Participants could in turn respond to the emotional pain/dissonance due to the loss of control. Similar to how the same young man did – by telling another person that he had become ill – it was possible to take responsibility by acknowledging the illness, and changing his behavior:

“*So I put the cards on the table and told my sister* ‘*Yes*, *I am gambling*-*addicted*!’. *It was extremely difficult*… *because people talk about you*… *like you are stupid*. *And I insist* -- *I*'*m not stupid*! *I had to defend myself*. *I told her*, *I*'*m not stupid*! *And she said she realizes this*. ‘*It*'*s a disease*.’ *So she was understanding*… *of it being a disease*, *and that it*'*s not something I can*… *that I wouldn*'*t have done this in a clear state of mind*. *And it meant a lot to me*, *that I wasn*'*t judged to be stupid*.”

The dissonance appeared emotionally painful, sometimes excruciating, including feelings of guilt, and a strong awareness of being personally responsible for the consequences related to the gambling. Despite the risk of losing the others' regard, participants would plan how to manage future temptations for the sake of righting their wrongdoings. For one man, problem acknowledgment had led him to the brink of suicide, having made an attempt less than three weeks before the interview. Undeniable consequences had made him consider the harms inflicted on his son due to his gambling behavior, and this had brought him into a depressive state, or a disillusioning, “empathic shock” that left him wanting to stay alive for his son's sake. This also became the solution to the hopeless situation:

“*I started thinking of my son*… *and I how much I love him*. *And it is no fun*, *having thoughts like that*. *So then I put all my cards on the table*, *for my family and my bosses*. *This was a very difficult acknowledgment to make*. *But I guess it was the easiest thing for me to do*. *So I*'*m dealing with the problem and I*'*ve come this far*: *I have told all the people who mean the most to me*.”

Resolving dissonance and moving towards a more positive self-image was an outcome of taking responsibility for the problem, as well as a constant source of motivation. For some participants, a positive identity was defined as “*a person taking responsibility for having a disorder*”, meaning it was acceptable to admit to being addicted to gambling if the participant were trying to change behavior. Maintaining a positive self-image was independent of the participants' sources of psychological pain, be it loss of money, social status, or empathy with negative implications of one's behavior on others, and motivational stage. For the participants who had recently sought treatment, the goal was to feel good about the self by taking responsibility and experiencing a sense of mastery. This meant being someone recognized by others as accountable for, and able to control, their actions:

“*I am happy and stably so*, *when I don*'*t gamble*. *Like now*, *I am happy with myself*. *I feel like I*'*m a* ‘*good kid*’… *who controls his finances*, *pays bills*… *works out*. *That is the first motivation*. *To be quite* ‘*normal*’.”

i. *Dissonance due to sports and athletics*.

For three of the five participants who had sought treatment, their motivation to change gambling included a desire to resume sports-related activities or being a sports fan, which were behaviors that had become personally stigmatized due their associations to gambling. This desire went along with planning leisure time and social activities as part of one's recovery. For example, one man had played soccer when younger, but then began betting on soccer games; beating problem gambling meant needing to find ways to partake in sports without betting:

“*It*'*s not easy*. *Some of the guys I play* (*soccer*) *with also gamble*. *But I have a family now*. *I don*'*t say that*, *but I think it*'*s implied*, *or something*. *I*'*ve signaled sufficiently that I can play with them*, *I just can*'*t gamble*.”

ii. *Dissonance due to gambling among family*

Gambling and problem gambling among family and concerned significant others also added to the perception of dissonance in a way that entangled their own motivations for changing with thoughts and feelings about these relationships. To proceed with their own problems, the participants also had to process the problems of their parents. Specifically, by comparing their parents' gambling problems to their own, participants could feel they were comparing themselves “downward”. That is, their family member was falling short of their own ideal self. This could reinforce both feelings of being special, such as being “lucky” or “good at gambling”, as well as feeling hopeless. A quote from one participant, shows how the mental image of his father's problems could boost his ego but simultaneously make him feel stuck:

“*I have a dad with the same problem*. *He*'*s gambling addicted*. *There*'*s no doubt*. *And he*'*s a really old man now*. *And always*, *ever since I was a child I*'*ve thought I wasn*'*t going to end up like dad*, *because it destroys so many families*, *so many relationships*. *He*'*s got it hard*, *we have it hard*. *And that doesn*'*t feel good*. *But I have to admit I*'*ve been going down the same track*. *And I*'*m actually going down it much quicker than him*… [*because of internet*] …. *Exactly*, *and with my access to capital*, *being a broker*… *my dad may not be as educated or smart*, *so he can*'*t abuse it the way I do*, *but I work with these things*, *I know how to get a loan*. *I know what to do to get most out of the different places*. *So it shows I*'*m clever*, *but what an unfortunate thing that I knew how to do*.”

## 4 Discussion

We identified two factors that supported the desire to minimize gambling. The first factor, *empathizing with the feelings of others*, is a psychosocial process based in part on empathizing with concerned significant others. The second factor is *dissonance between gambling behavior and ideal self*-*image*, which describes a cognitive and emotional appraisal of the consequences of the gambling relative to past and future versions of oneself. The latter process included two identity-based subthemes in which ideal selves, including previously held personal skills in sports/athletics and the idea of having a well-functioning family of significance, became discordant in the context of gambling problems.

### 4.1 Sources of dissonance and motivation

In the process of transitioning from external to internal motivation we observed that motivation for change arises from negative consequences, producing a basic motivation to restore psychosocial and financial equilibrium to obtain a positive self-feeling. This process of becoming motivated from dissonance is commonly described within self-discrepancy theory (Higgins, 1998). The dissonance between the perception of ideal and actual behaviors produced a recognition of the impulse to gamble as something bad, involving feelings of guilt and shame, as well as an assessment of “not being normal”. The process of dissonance included the subthemes of *sports and athletics* and *familial gambling*. Participants talked about the difficulties of finding new ways to relate socially through sports and athletic activity while changing gambling habits. Some also discussed intergenerational gambling problems within their families, and described difficulties in coming to terms with their own gambling due to the fear of additionally stigmatizing or letting down another family member. Previous positive social identities, connected to sports and athletics, were described as tarnished due to their associations with gambling problems. Likewise, knowing that older family members, with whom the participants could relate and empathize, also have gambling problems, was seen as a challenge to esteem, causing dissonance. These findings are in line with studies indicating that problem gambling is more commonly developed in individuals who strongly identify with playing sports. Although none of our participants had ever played sports professionally or in college, a majority (*n* = 11) had been actively involved with athletics throughout high school and later. This association to athletics means they may have had a higher exposure to gambling. Having a history of problem gambling within the family caused participants to place an even greater responsibility upon themselves to spare their family from further suffering.

### 4.2 Social process of empathy, goal attainment and projections

The findings of this study also indicated that shifts in participants' reported motivation for change occurred in the context of exploring negative effects on concerned significant close, and/or distant others. This was indicated by identifying thoughts about how significant others would feel about the participant's gambling and related consequences, not acknowledging personal addiction directly, but indirectly by acknowledging that “family members struggled with addiction as well”. The gamblers were likely able to deny having these feelings until faced with either the feelings of a close or distant significant other. Insights into the minds of significant others were not restricted to what the significant others actually had expressed, but also included the participants' own hypotheses about what might be experienced by significant others. Projections are primarily known as defense mechanisms (Baumeister, Dale, & Sommer, 1998). They constitute unconscious fantasies attributed to an external object so to get rid of aspects of one's own psyche. That is, the act of perceiving in other people the characteristics that you do not like in yourself (Newman, Duff, & Baumeister, 1997). In this case, participants projected negative feelings about their gambling habits. While defense mechanisms can be viewed as obstacles to treatment, they can also facilitate self-exploration. In the present study they provided some of the gamblers an opportunity to safely feel and express negative emotions connected with their gambling habits, as if they were seeing the behavior through the eyes of a hypothetical or real, but unaware, concerned significant other.

### 4.3 Implications

The finding that projections may induce motivation for change suggests that clinicians or e-health interventions may use projections to explore dissonance within this group, for example by soliciting users' imagining significant others' thoughts and feelings about their gambling habits. Most current gambling interventions, whether they include face-to-face counseling or online programs, focus on eliciting the user's thoughts and feelings regarding negative consequences due to gambling (Rizeanu, 2015). Trained therapists may become aware of, and use induced projections to help patients recognize, act on, and re-attach meanings to their feelings (Johansen, Tavakoli, Bjelland, & Lumley, 2016). It may be seen as an indirect approach to exploring the person's own thoughts and feelings, but as it is presented as focusing on the significant other, it generates less resistance, aligning it with approaches such as Motivational Interviewing (Miller & Rollnick, 2013). When actual close significant others are highly critical, there may be an advantage to utilizing fictional characters to prompt helpful projections about problem gambling. It might make a difference whether the individual feels that the comparison with a significant other is made to criticize or help them. It is possible that shame and guilt experienced by the gambler after gaining insights into their behaviors may reduce ongoing help-seeking behaviors (Gainsbury, Hing, & Suhonen, 2014). When designing therapeutic elements to support projections it is therefore important to be mindful of whether the significant other used to solicit the projection is perceived as supportive, or potentially critical of the user.

Our advice to focus on significant others' is in line with research into health communication message tactics that has shown that focusing on consequences directed at significant others may be more effective in motivating for change than focusing on the individual (Keller & Lehmann, 2008). User support needs are time-sensitive (Hupcey, 1998), meaning it is possible that not all users of e-health programs utilizing projective motivational features will benefit from the inclusion of these features. Based on these results, we would expect that efforts to normalize the commonalities of inherited and intergenerational gambling problems are motivating when framed as intended to relieve psychological pain. Additionally, it may be possible to build hope by providing people with stories of users' successful disentanglement of their passion for sports and gambling.

### 4.4 Limitations

The study was conducted with a small sample, using cross-sectional, qualitative interviewing as the main method. Findings may therefore not generalize to individuals who gamble in all settings. For example, while we found comorbid substance use to be part of the dissonance of one of the treatment-seeking participants, the theme did not stand out as a subtheme in its own right. This may be because our sample included a larger number of non-treatment-seeking than treatment-seeking participants. It may be that a larger sample of individuals already motivated to change their behavior would have produced a greater recognition of comorbid substance abuse problems. Executing interviews within the participants' homes and places of work may influenced participants in terms of catering their responses towards social desirability, or may have influenced their sense of confidentiality in the results. Further research into motivation for change of problem gambling thus needs to explore the narratives of populations not represented in this study, including females and ethnic and cultural minorities, and using alternative research methods from that of the current study.

### 4.5 Summary and conclusions

The current study showed that motivation for change of gambling behaviors was an outcome of empathizing with the feelings of close and distant others and exploring dissonance. Personal motivations towards sports and athletics, and the potential effects of inherited gambling problems including the guilt of concerned significant others who have their own concerns about gambling or addiction may add value to current conceptualizations of motivation in problem gambling. In the context of e-health behavior change programs, the dissonance subthemes may most easily be construed as a series of case vignettes, showing users who have successfully coped with their ambivalence due to family issues or sports. As indicated by this study, however, projections based on reactions to materials of hypothetical close others, such as the “girlfriend character” used in this study, may benefit motivation for change when close others are critical, and/or the user fears conflict and confrontations at home. To ensure successful, positive effect, as well as enable measurement of the specific effect size involved with using projective motivational features in e-health programs, future studies must attend to the perceived framing of users' projections to make sure they add to, rather than subtract from treatment. Specifically, as facilitating projections seeks to destabilize motivation temporarily in order to strengthen it, there is also a risk of demotivation. Such disruption to building motivation must be mended through an active process of assessing and re-attributing meaning to the user reactions. Escape from life problems is a characteristic of problem gambling (Wood & Griffiths, 2007), and shame and guilt may reduce help-seeking. Therefore, the use of projections to internalize motivation involves a risk of detrimental effects and must be used with caution, particularly in the context of non-guided online interventions. Future studies into the use of projective motivational features used to motivate online problem gamblers should assess both users' family and relationship histories concerning gambling, as well as how they perceive the framing of the feelings of close others.

## Declaration of Competing Interest

The authors report no conflicts of interest.
